# Metal-Free Synthesis of *N*-Heterocycles *via* Intramolecular Electrochemical C-H Aminations

**DOI:** 10.3389/fchem.2022.950635

**Published:** 2022-06-20

**Authors:** Huiqiao Wang, Yongjun Zheng, Hucheng Xu, Jiaru Zou, Congrui Jin

**Affiliations:** ^1^ School of Chemical and Environmental Engineering, Anyang Institute of Technology, Anyang, China; ^2^ Department of Civil and Environmental Engineering, University of Nebraska–Lincoln, Lincoln, NE, United States

**Keywords:** organic electrosynthesis, C-H amination, N-heterocycle, metal-free, cyclization, N-centered radical

## Abstract

*N*-heterocycles are key structural units in many drugs, biologically interesting molecules and functional materials. To avoid the residues of metal catalysts, the construction of *N*-heterocycles under metal-free conditions has attracted much research attention in academia and industry. Among them, the intramolecular electrochemical C-H aminations arguably constitute environmentally friendly methodologies for the metal-free construction of *N*-heterocycles, mainly due to the direct use of clean electricity as the redox agents. With the recent renaissance of organic electrosynthesis, the intramolecular electrochemical C-H aminations have undergone much progress in recent years. In this article, we would like to summarize the advances in this research field since 2019. The emphasis is placed on the reaction design and mechanistic insight. The challenges and future developments in the intramolecular electrochemical C-H aminations are also discussed.

## Introduction

*N*-heterocycles are key structural units in many drugs, biologically interesting molecules and functional materials (Kim and Movassaghi, 2009; Meng et al., 2021; Staśkiewicz et al., 2021). Therefore, the development of robust synthetic methods for the construction of *N*-heterocycles has gained increased attention in academia and industry. Over the past decades, many metal-catalyzed methodologies such as Buchwald-Hartwig cross coupling (Newman and Lautens, 2010) and Ullmann reactions (Lu and Li, 2006) have provided direct routes to obtain *N*-heterocycles with high efficiencies. They are indeed one of the best choices for the preparation of *N*-heterocycles in laboratory. However, the difficulty to remove the metal residues resulted from the metal catalysts largely limits the industrial use of these metal-catalyzed methodologies. To circumvent this limitation, many metal-free methodologies have been developed for the synthesis of *N*-heterocycles recently (Preeti and Singh, 2021; Yang et al., 2021; Han et al., 2022; Yamamoto et al., 2022). Among them, the intramolecular C-H aminations are of high interest mainly because the prefunctionalized substrates are avoided (Tan et al., 2020). In light of the significance of *N*-heterocycles, the development of atom economy and environmentally benign methods for intramolecular C-H aminations continues to play a pivotal role in medicinal and material chemistry.

Organic electrosynthesis utilizes electricity as the driving force to activate substrates of interest, thus offering a sustainable way to obtain reactive intermediates for organic synthesis (Li et al., 2019; Meyer et al., 2020; Luo et al., 2021; Novaes et al., 2021; Chen et al., 2022; Qian et al., 2022; Tan et al., 2022; Wang and Xu, 2022). Recently, organic electrosynthesis has undergone considerable renaissance, and provided unique reactivities that are not accessible with traditional synthetic methodologies. With these benefits, organic electrosynthesis has widely been employed in the construction of C-C (Pollok and Waldvogel, 2020; Zhang et al., 2021), C-O (Herszman et al., 2019; Zhang et al., 2019), C-S (Zhang et al., 2020) and C-Si (Ke et al., 2021; Jiang et al., 2022) bonds in green manners. In the context of intramolecular C-H aminations, organic electrosynthesis has also showed great synthetic potentials. Many types of *N*-heterocycles were electrochemically synthesized under metal- and external oxidant-free conditions. Compared with the traditional metal-free methods for *N*-heterocycles syntheses, these electrochemical protocols feature environmentally friendly conditions and operational simplicity. In this mini-review, would like to summarize the advances in intramolecular electrochemical C-H aminations under metal-free conditions since 2019. The contents were categorized by the C (sp^2^)-H and C (sp^3^)-H aminations. Our focus is on both 1) reaction design including electrolytic conditions, catalyst choice and substrate design, and 2) mechanistic insights of intramolecular C-H aminations. Finally, the challenges and future developments in this research field are also discussed. We hope this mini-review will offer a balanced overview of the recent contributions to intramolecular electrochemical C-H aminations, and will act as a useful reference to students and scientists working in this research field.

## Intramolecular Electrochemical C(sp^2^)-H Aminations

**SCHEME 1:**
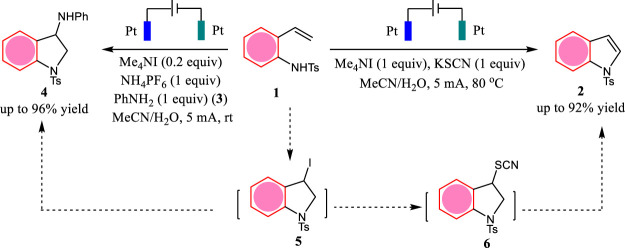
The electrochemical synthesis of indoles and indolines.

Indoles and indolines are widely existed in many biologically important molecules (Newhouse and Baran, 2008). In 2020, Wang and co-workers report an iodide-mediated electrochemical C (sp^2^)-H amination methodology for the tunable synthesis of indoles **two** and indolines **4** (Scheme 1) (Hu et al., 2020). This tandem reaction was proposed to proceed *via* the key intermediate **5**. When the reaction was carried out in the presence of KSCN, the nucleophilic substitution of iodide by KSCN gives intermediate **6**. The *β*-H elimination within **6** affords indoles **2**. However, when the reaction was carried out in the presence of PhNH_2_ (**3**), the nucleophilic substitution of iodide by PhNH_2_ directly yields indolines **4**. This protocol features metal- and external oxidant-free conditions and switchable synthesis of indoles and indolines.

**SCHEME 2:**
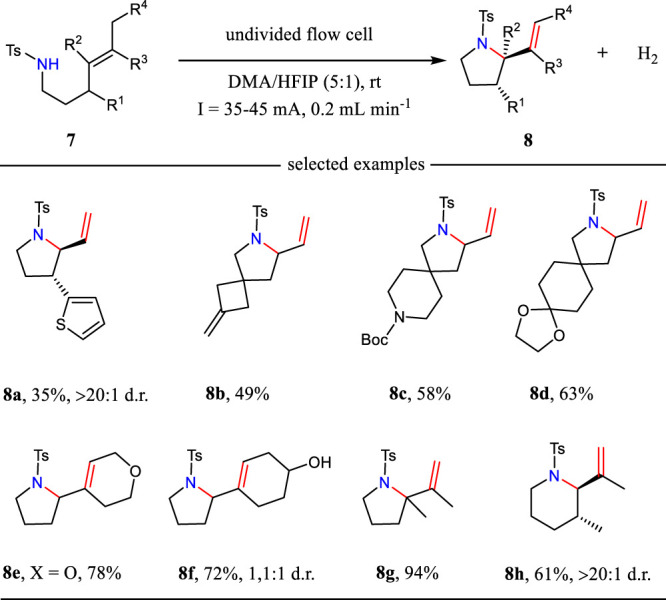
The formal aza-Wacker cyclizations under continuous-flow electrochemical conditions.

Aza-Wacker cyclization is one of the most straightforward methods to synthesize alkene-functionalized *N*-heterocycles. The traditional methods often relied on the Pd catalysis, thus leading to unexpected metal residues (Thomas et al., 2020). In 2021, Xu and co-workers report a new formal electrochemical aza-Wacker cyclization under continuous-flow conditions (Huang et al., 2021) (Scheme 2). This reaction was carried out with dimethylacetamide (DMA) and 1,1,1,3,3,3-hexafluoro-2-propanol (HFIP) as the mixed solvents. This newly developed protocol allows the construction of saturated *N*-heterocycles with up to 94% yield. The catalyst- and supporting electrolyte-free conditions make this electrochemical method more appealing to the developed methodologies. Moreover, this electrochemical condition tolerates multiple substituted alkenes well, which are challenging substrates in metal-catalyzed aza-Wacker cyclizations.

**SCHEME 3:**
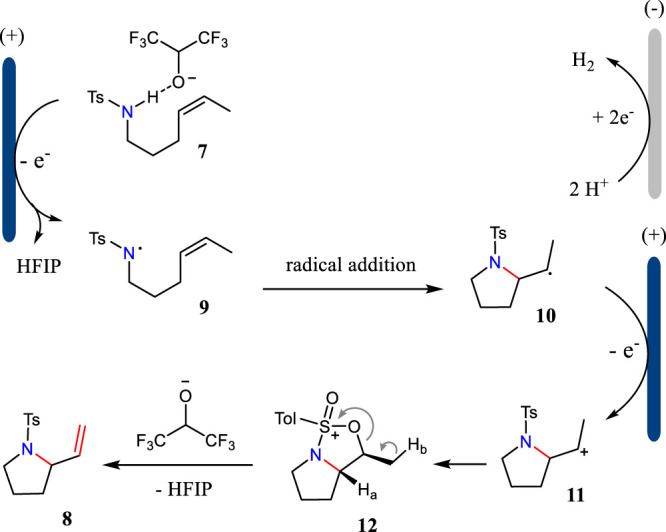
The tandem reaction sequences for the aza-Wacker cyclizations.

The tandem reaction sequence for the aza-Wacker cyclizations is shown in Scheme 3. First, the substrate has a SET oxidation at the anode *via* a proton-coupled electron-transfer (PCET) pathway to give *N*-centered radical **9**. Then, radical **nine** undergoes intramolecular radical addition to alkene leading to the formation of radical **10**, which has a further SET oxidation to yield intermediate **11**. The intramolecular cyclization of intermediate **11** gives bicyclic cation **12**, which has an elimination reaction to afford the desired product **8**. The electrochemically generated hexafluoroisopropoxide plays an important role to drive the reaction.

**SCHEME 4:**
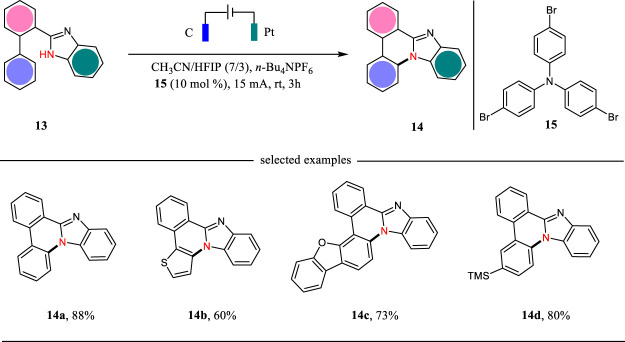
The electrochemical synthesis of benzimidazole-fused phenanthridines.

Benzimidazole-fused phenanthridines are excellent candidates as fluorescent and phosphorescent organic light-emitting diodes (Ohsawa et al., 2019). The classic methods for the synthesis of benzimidazole-fused phenanthridines relied on the Pd catalysis or hypervalent iodine oxidation reactions. In 2021, Xu, Zhang and co-workers report a triarylamine-mediated electrochemical protocol for the formation of benzimidazole-fused phenanthridines in good to excellent yields (Shi et al., 2021) (Scheme 4). The reaction was carried out in an undivided cell with carbon cloth as the anode and platinum as the cathode under CCE conditions. This reaction was proceeded through an intramolecular C-H amination sequence with hydrogen evolution as the side reaction. For some substrates, the direct electrolysis was also efficient to facilitate the intramolecular C-H amination reactions.

**SCHEME 5:**
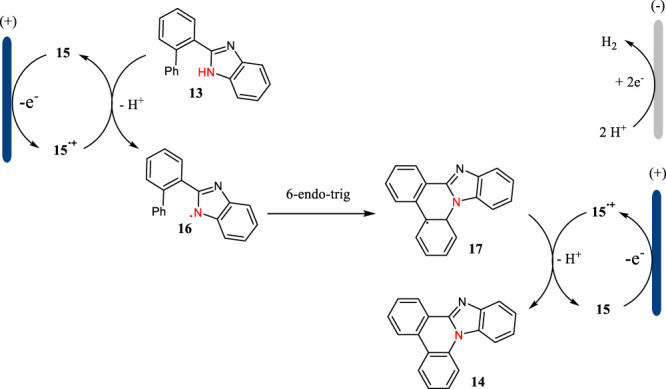
The plausible mechanism for the triarylamine-mediated C-H aminations.

The plausible mechanism for the triarylamine-mediated C-H aminations is shown in Scheme 5. Triarylamine **15** acting as an organic mediator has a SET oxidation at the anode to give the corresponding radical cation, which then undergoes an electron transfer with **13** to afford *N*-centered radical **16**. The radical **16** could have a *6-endo-trig* cyclization to give intermediate **17**, which then undergoes an indirect oxidation followed by proton releasing to yield benzimidazole-fused phenanthridine **14**. Simultaneously, the proton is reduced to hydrogen gas at the cathode.

**SCHEME 6:**
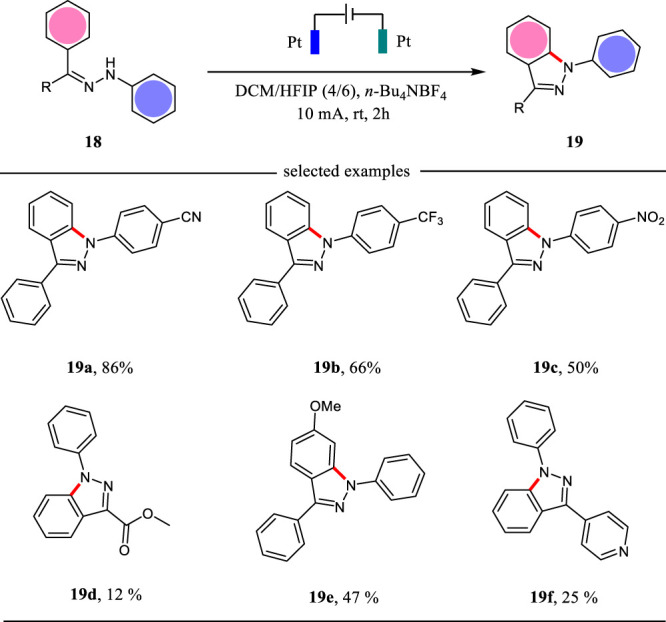
The intramolecular electrochemical cyclization for the synthesis of 1H-indazoles.

1H-indazoles as one important class of *N*-heterocycles showed interesting biological activities (Schmidt and Dreger, 2011). The reported methods for the synthesis of 1H-indazoles are limited by the use of metal catalysts at high temperature or stoichiometric chemical oxidants such as Oxane. To circumvent these limitations, Lei and co-workers developed an electrochemical strategy for the synthesis of 1H-indazoles under metal-free conditions (Wan et al., 2022). This intramolecular cyclization was carried out in an undivided cell with platinum plates as the electrodes and *n*-Bu_4_NBF_4_/DCM/HFIP as the electrolyte solution under CCE conditions. As shown in Scheme 6, a variety of 1H-indazoles were obtained in good to excellent yields. The functional groups such as cyano and ester were tolerated well under the optimal conditions. This reaction represents the first example to construct 1H-indazoles under metal- and external-oxidant conditions.

**SCHEME 7:**
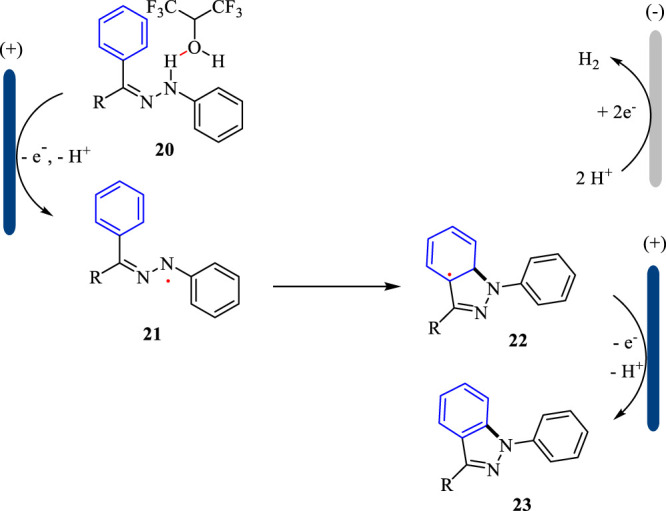
The plausible mechanism for the electrochemical synthesis of 1H-indazoles.

The plausible mechanism for the electrochemical synthesis of 1H-indazoles is shown in Scheme 7. With the assistance of HFIP, the substrate **20** is oxidized at the anode and loses one molecule of proton giving *N*-centered radical **21**, which undergoes the following intramolecular cyclization to afford radical **22**. Subsequently, the intermediate **22** undergoes another SET oxidation followed by deprotonation to yield 1H-indazole **23**. Simultaneously, the proton is reduced to hydrogen gas at the cathode. The hydrogen-bond interaction between HFIP and substrate **20** is the key to the success of the intramolecular cyclization reaction.

**SCHEME 8:**
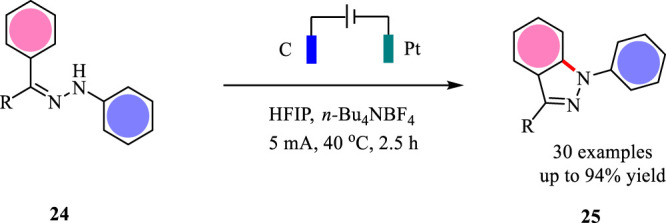
The electrochemical C-H/N-H coupling for the synthesis of 1H-indazoles.

**SCHEME 9:**
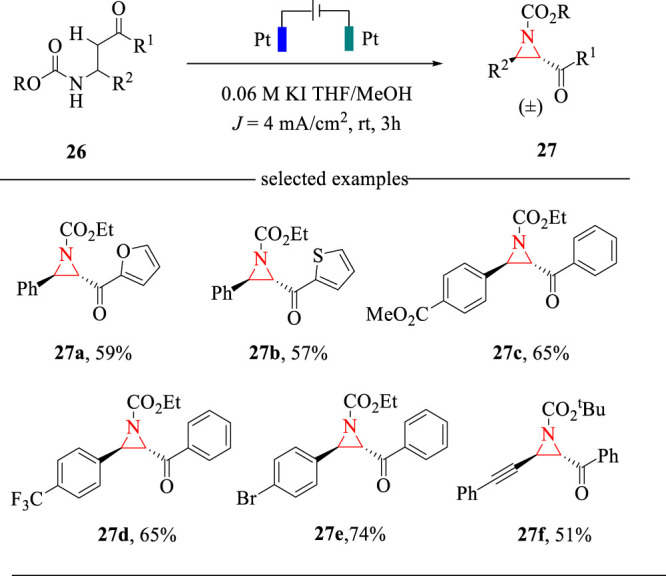
The electrochemical synthesis of aziridines.

At almost the same time, Zhang’s group reported a similar electrochemical protocol for the formation of 1H-indazoles *via* an intramolecular C-H/N-H coupling reaction (Zhang et al., 2022) (Scheme 8). Different from Lei’s protocol, this electrochemical protocol employs graphite rod as the anode and HFIP as the solvent. As shown in Scheme 9, the desired 1H-indazoles were obtained with up to 94% yield.

## Intramolecular Electrochemical C(sp^3^)-H Aminations

Aziridines are key structural units in many bioactive natural products, thus attracting much attention towards their facile syntheses (Miyazaki et al., 2015). The classical methods relied on the cycloaddition strategy to access aziridines (Degennaro et al., 2014). However, the expensive nitrene or carbene precursors and harsh reaction conditions are always needed. Encouraged by recent progress on halide-mediated electrochemical transformations (Lian et al., 2021) and our effort on the electrochemical cyclization for the synthesis of 2-oxazolines (Wang et al., 2018), we recently developed a KI-mediated intramolecular C-H amination reaction for the synthesis of *trans*-2,3-disubstituted aziridines (Wang et al., 2019) (Scheme 9). The electrochemical cyclization reaction was carried out in an undivided cell with Pt plate as the electrodes and THF/MeOH as the mixed solvents. This intramolecular C-H amination reaction allows the construction of 2,3-disubstituted aziridines in moderate yields with exclusively *trans* selectivities. This method also represents the first example of electrochemical synthesis of aziridines *via* intramolecular C-H amination strategies.

**SCHEME 10:**
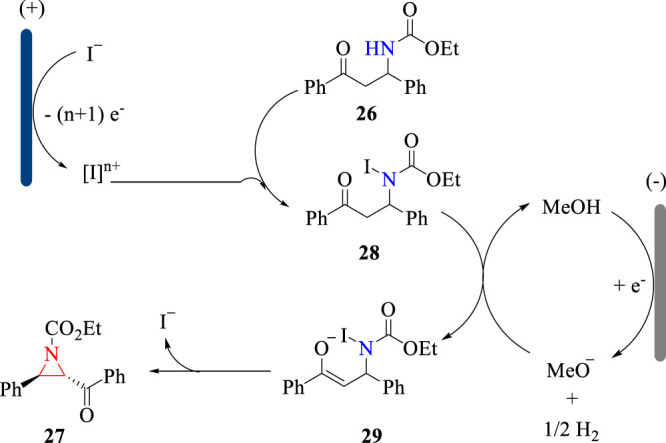
The reaction sequence for the electrochemical formation of aziridines.

The electrochemical C-H amination reaction was proposed to proceed *via* the oxidative reaction sequence. As shown in Scheme 10, KI is oxidized to hypervalent iodine, which undergoes an iodination reaction with substrate **26** to afford intermediate **28**. Then, intermediate **28** is deprotonated by electrogenerated MeO^−^ to deliver intermediate **29**, which undergoes the intramolecular cyclization to give aziridine **27** in a *trans* selectivity. The electrogenerated MeO^−^ acts as a strong base to drive the cyclization reaction and thus obviating the addition of external bases.

**SCHEME 11:**
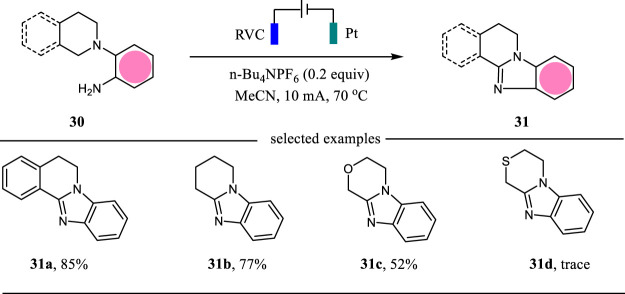
The electrochemical synthesis of benzimidazoles.

Benzimidazoles are valuable *N*-heterocycles possessing important biological and pharmacological activities (Hegde et al., 2015). The classical methods to access benzimidazoles relied on the condensation reaction at high temperatures. The intramolecular C-H amination reactions need Ir catalyst or 3-chloroperbenzoic acid (*m*-CPBA) as the oxidant (Sun et al., 2015). In 2019, Tang, Pan and co-workers reported an electrochemical C (sp^3^)-H amination protocol for the construction of benzimidazoles in moderate to excellent yields (Li A. et al., 2021) (Scheme 11). This intramolecular C-H amination reaction was carried out in an undivided cell with RVC as the anode and Pt as the cathode and CH_3_CN as the solvent. It is noteworthy that this electrochemical protocol showed an excellent functional group tolerance.

**SCHEME 12:**
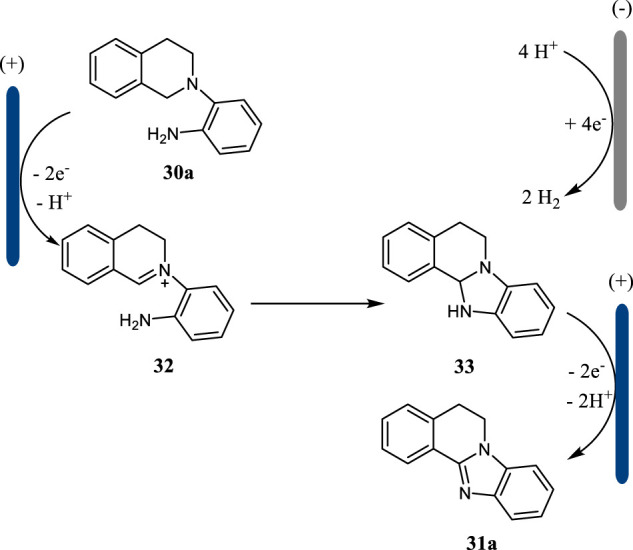
The reaction mechanism for the formation of benzimidazoles.

This intramolecular C (sp^3^)-H amination was proposed to proceed *via* an intramolecular oxidative cyclization mechanism (Scheme 12). The substrate **30a** undergoes a two-electron oxidation followed by deprotonation to give imine **32**, which then cyclizes to afford intermediate **33**. The further anodic oxidation of intermediate **33** followed by deprotonation lead to the formation of benzimidazole **31a**. Simultaneously, the proton is reduced to hydrogen gas at the cathode. This intramolecular C (sp^3^)-H amination strategy may find more applications in the synthesis of other types of *N*-heterocycles.

**SCHEME 13:**
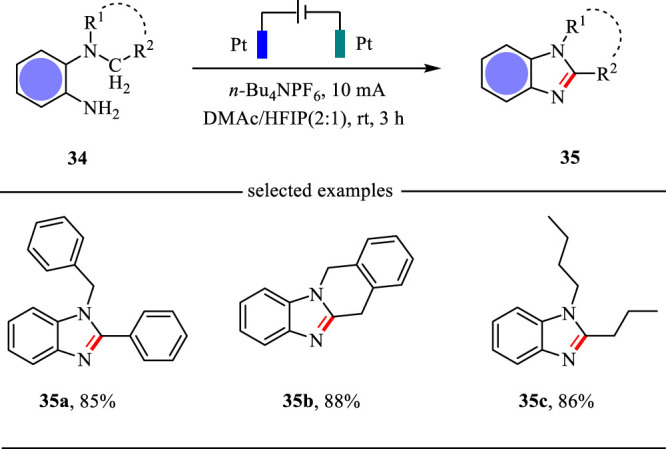
The electrochemical synthesis of benzimidazoles.

Following the similar reaction mechanism, Tang, Zhou and co-workers reported an electrochemical C (sp^3^)-H amination strategy for the construction of benzimidazoles in excellent yields (Li J. et al., 2021) (Scheme 13). This intramolecular C-H amination reaction was carried out in an undivided cell with Pt as the electrodes and *N*, *N*-dimethylacetamide (DMAc)/HFIP as the mixed solvents. This electrochemical strategy features mild conditions and wide substrate scope.

## Summary and Outlook

In recent years, the intramolecular electrochemical C-H aminations have emerged as powerful synthetic tools for the synthesis of *N*-heterocycles under metal-free conditions. This mini-review highlights the recent contributions to the field of metal-free synthesis of *N*-heterocycles *via* intramolecular electrochemical C-H aminations since 2019. The related contents were categorized by the C (sp^2^)-H and C (sp^3^)-H aminations. The detailed mechanisms for the C-H aminations were discussed to shed light on the reaction design principle for these electrochemical transformations. Although significant advances in this research field have been made, some challenges persist. First, large amounts of supporting electrolytes are necessary for the reported intramolecular electrochemical C-H aminations. Considering the trend of green synthesis, the use of continuous-flow electrochemistry under supporting electrolyte-free conditions is one of the directions in this domain. Second, compared with the well-established intramolecular electrochemical C (sp^2^)-H aminations, C (sp^3^)-H aminations only have limited successful examples. The development of new strategies based on intramolecular electrochemical C (sp^3^)-H aminations would provide new opportunities to access *N*-heterocycles of interest. This field will soon find more opportunities in the synthesis of functional materials and drugs on academic and even industrial level.
